# Supplementary material for: Phosphatidylserine Receptors Enhance SARS-CoV-2 Infection: AXL as a Therapeutic Target for COVID-19
Source: bioRxiv. 2021 Jun 15:2021.06.15.448419. Preprint. [Version 1] doi: 10.1101/2021.06.15.448419 (PMC8219095; doi:10.1101/2021.06.15.448419)
Supplement: Supplementary file 1 [file media-1.pdf]

S1: Supplemental data related to Figure 1

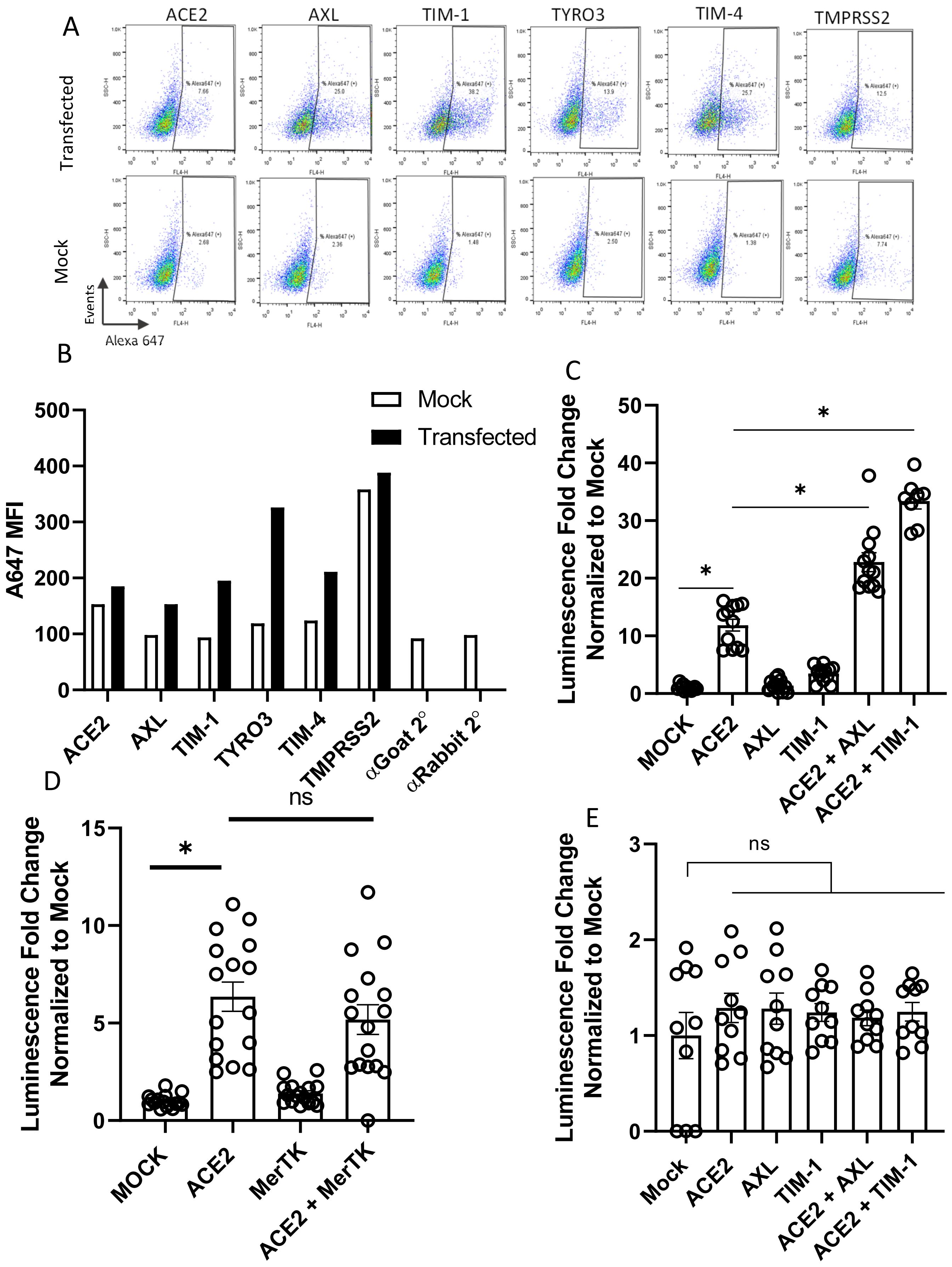

Supplemental Figure 1: PS receptors synergize with ACE2, enhancing SARS-CoV-2 infection of HEK 293T cells. **A)** Representative surface staining of receptors transfected into cells. **B)** Surface expression (MFI) of proteins in mock transfected (empty vector) and transfected HEK 293T at 48 hours after transfection**.** Background fluorescence is shown for secondary antibodies used in experiment (α-goat or rabbit secondaries). **C)** HEK 293T cells, transfected PS receptors as noted with or without 250 ng of ACE2 were transduced with rVSV-luciferase/Spike. Transduction was assessed 24 hours later via luminescence. **D)** Expression of MerTK did not affect rVSV-luciferase/Spike transduction in the presence of 250 ng of transfected ACE2 plasmid. **E)** Expression of ACE2, TIM-1 or AXL did not enhance infection of VSV-luciferase/Lassa virus GP pseudovirions. HEK 293T cells were transfected with PS receptor plasmids and 50 ng of ACE2 and infected 48 hours later**.** Panels C-E are shown as fold change of luciferase activity in cell lysates relative to mock transfected lysates that were set to a value of 1.

Data shown are pooled from at least three independent experiments (**C**, **D**, and **E)**. Data represented as means ± SEM. One-Way ANOVA with multiple comparisons (**C, D**), Student's t-test (**E**); asterisks represent $p < 0.05$.

S2: Supplemental data related to Figure 2

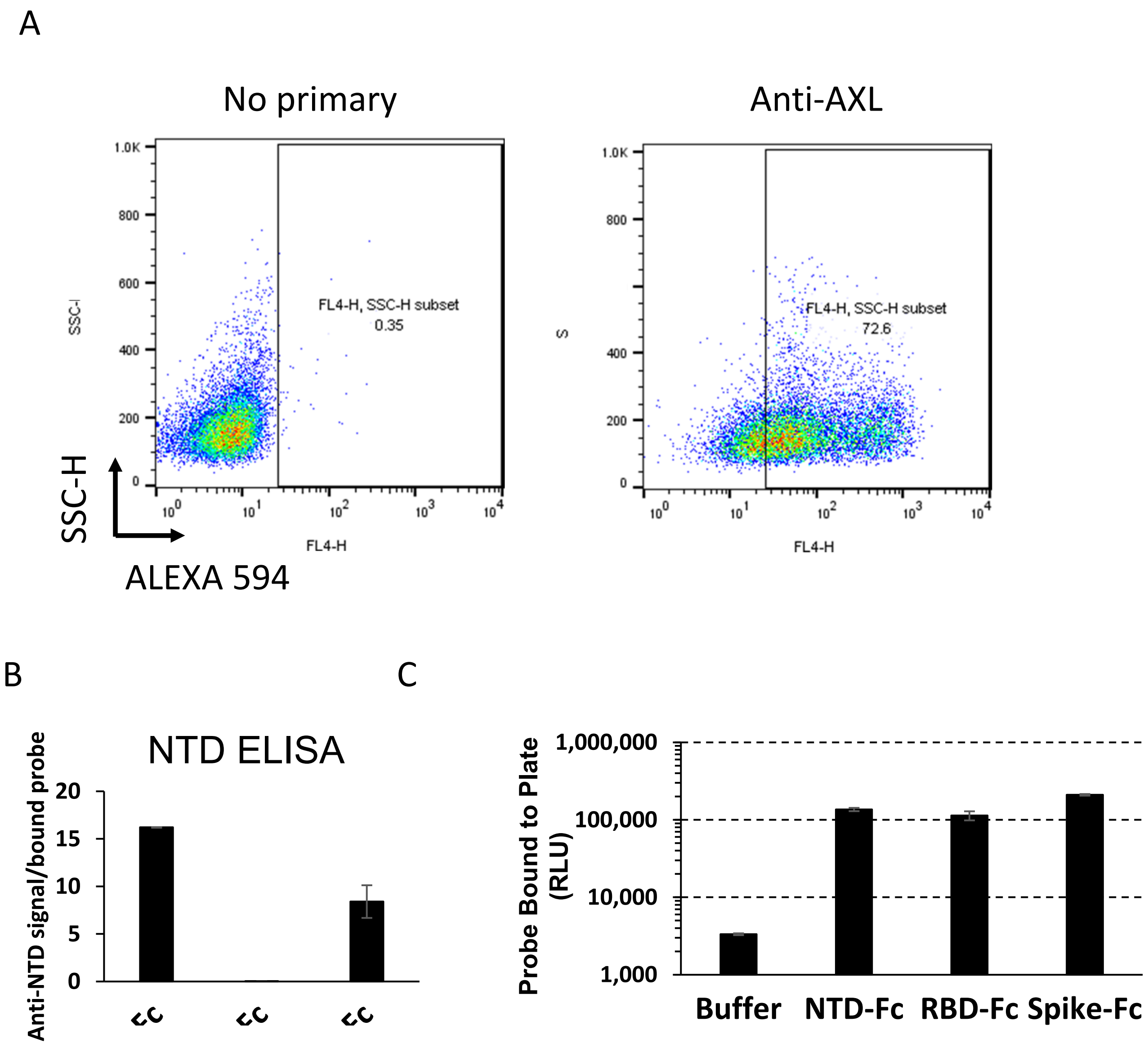

Supplemental Figure 2: PS receptors interact with SARS-CoV-2 by binding to PS. **A**) AXL surface expression in transfected HEK 293T cells. **B**) Soluble purified S1/S2-Fc and NTD-Fc are detected by an NTD monoclonal antibody by ELISA. **C**) All spike-Fc proteins bind and are detected at equivalent levels of ELISA plates.

S3: Supplemental data associated with Figure 3

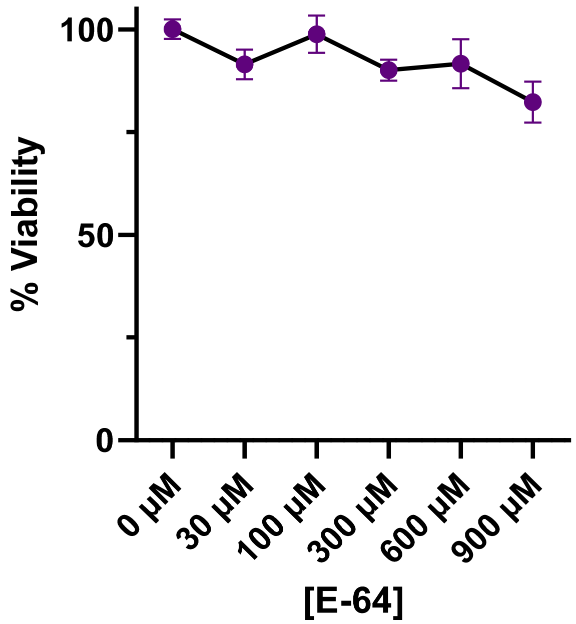

Supplemental Figure 3: The route of SARS-CoV-2 entry is altered by TMPRSS2 expression. ATPLite cytotoxicity assay in H1650 cells, 24 hours following treatment with E64. Data are represented as means +/- SEM.

S4: Supplemental data associated with Figure 4

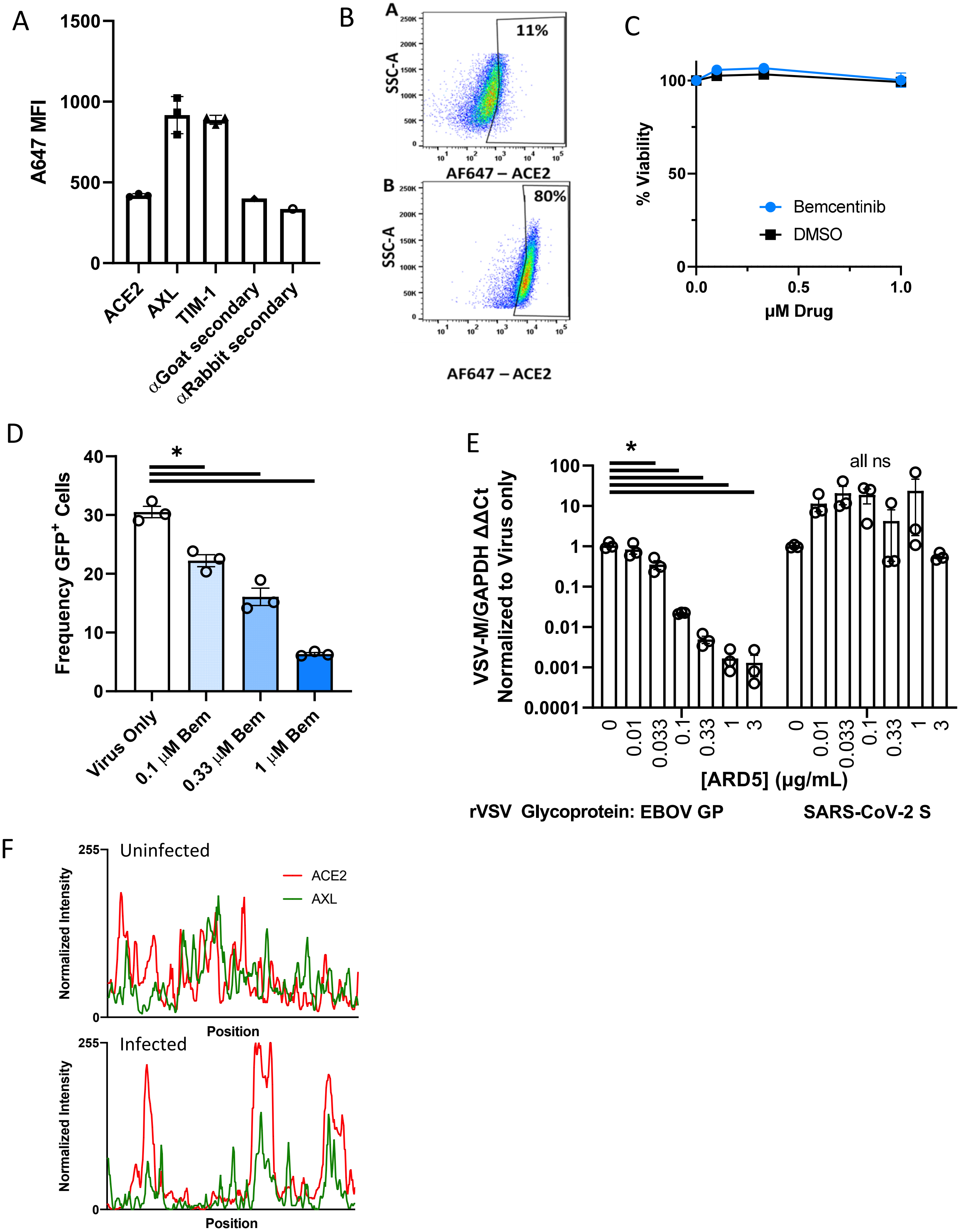

Supplemental Figure 4: AXL has a prominent role in SARS-CoV-2 entry in Vero E6 cells. **A)** ACE2, AXL and TIM-1 surface expression MFI in Vero E6 cells, as assessed by flow cytometry. Background fluorescence is shown for secondary antibodies used in experiment. **B)** Cell surface versus intracellular ACE2 expression in VeroE6 cells. Indicated cells were lifted, permeabilized as noted, and stained with anti-ACE2 unconjugated primary antibodies and Alexa 647 secondaries. **C)** Bemcentinib toxicity 24 hours after treatment was measured by ATPlite assay in H1650 cell line. **D)** VSV-GFP/Spike entry was measured by flow cytometry 24 hours after challenge to Vero E6 cells treated with bemcentinib. **E)** Vero E6 were treated with ARD5 (TIM-1 blocking antibody) 1 hour before infection with rVSV /SARS-CoV-2 Spike or rVSV /EBOV-GP (MOI = 0.01). Viral load was measured 24 hpi by RT-qPCR. **F)** Plot profiles of ACE2 and AXL intensity are shown in from STED micrographs in Fig. **4G**, representing signal intensity along the yellow lines in the merged panels.

Data in A, C, D and E are shown as means ± SEM. Multiple t-tests were performed in C and Student's t-test was performed in D and E; asterisks represent $p < 0.05$.

.

S5: Supplemental data related to Figure 5

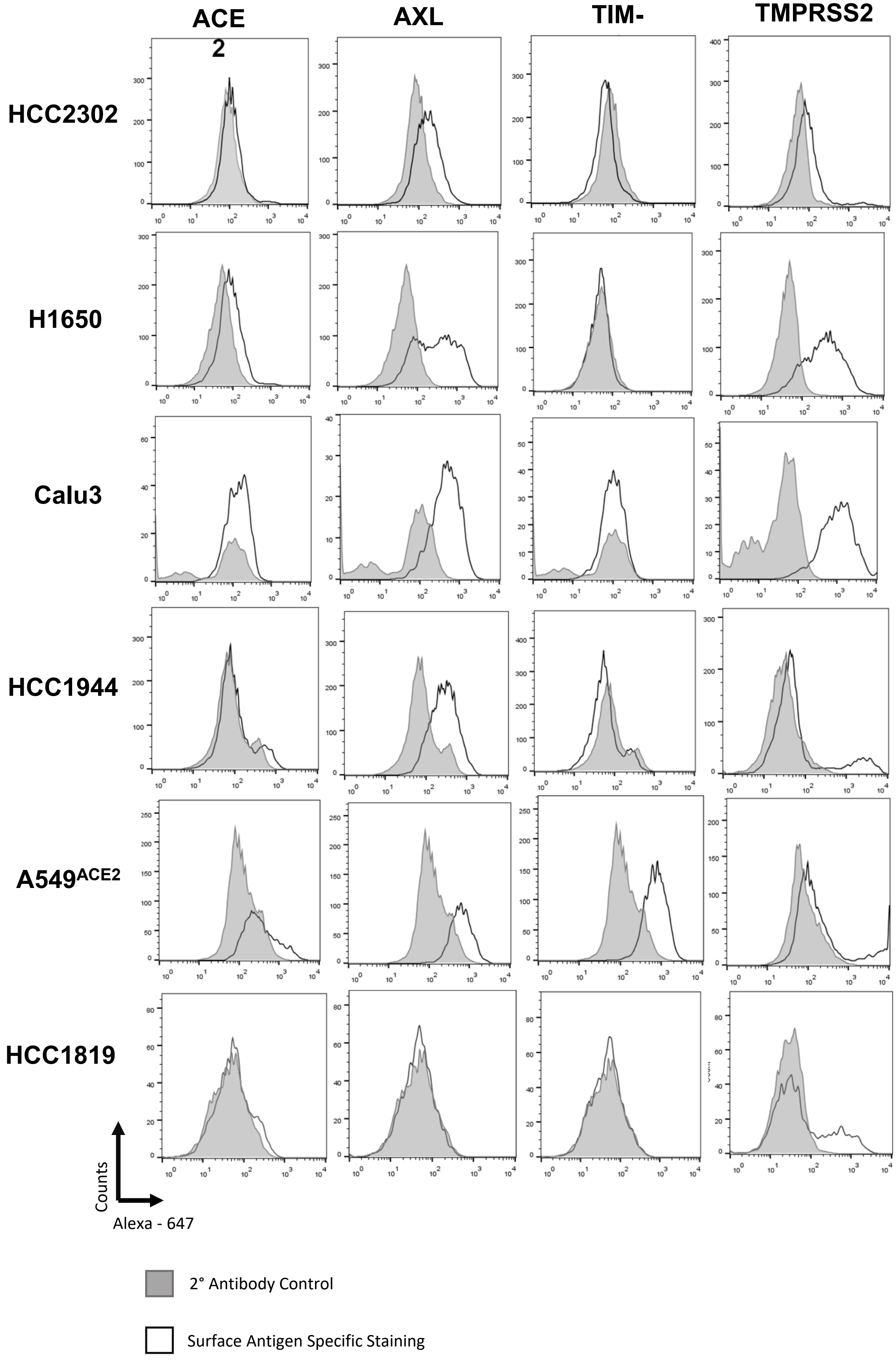

S5 con't: Supplemental data related to Figure 5

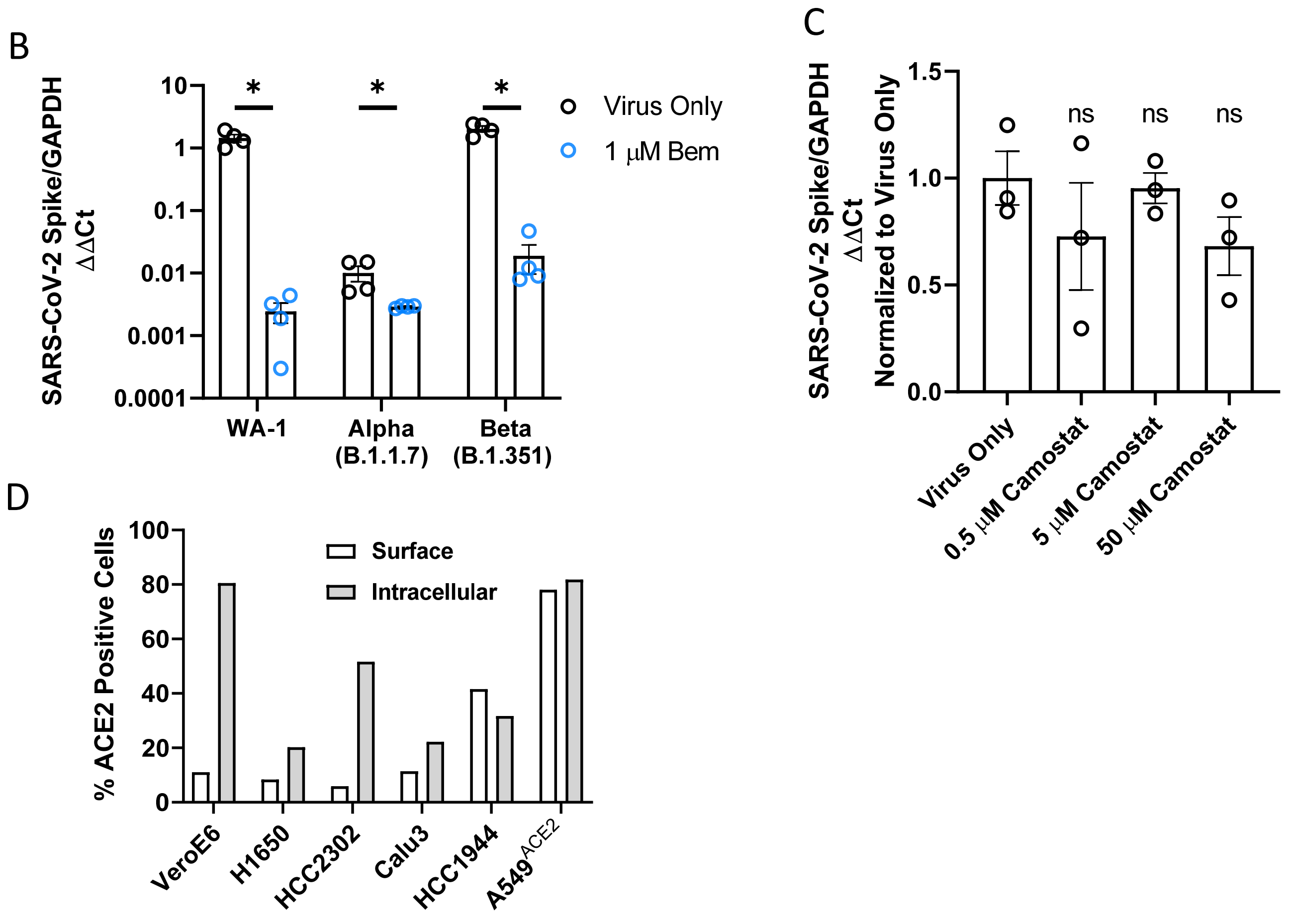

Supplemental Figure 5: AXL inhibition reduces SARS-CoV-2 infection in human lung cells. A) Multiple SARS-CoV-2 permissive cell lines were stained for extracellular ACE2, AXL, TIM-1, and TMPRSS2 protein, and expression was quantified by flow cytometry. Shown are flow cytometry histograms depicting target surface staining (black line) and secondary only background (grey shade) **B)** H1650 cells were treated with 1 μM of bemcentinib and infected with one of three different variants of SARS-CoV-2: WA-1; B.1.1.7 or B.1.351 (MOI=0.5 for all variants). RNA was isolated at 24 hpi and assessed for virus load. **C)** H1650 cells were infected with SARS-CoV-2 (MOI = 0.5) after treatment with the indicated concentration of camostat for 1 hour. Viral loads 24hpi were measured by qRT-PCR. **D)** Extracellular and intracellular staining of ACE2 are shown in multiple cell lines. Presented as frequency positive cells.

Data represented as means ± SEM. Student's t-test; asterisks represent $p < 0.05$.

S6: Supplemental data related to Figure 6

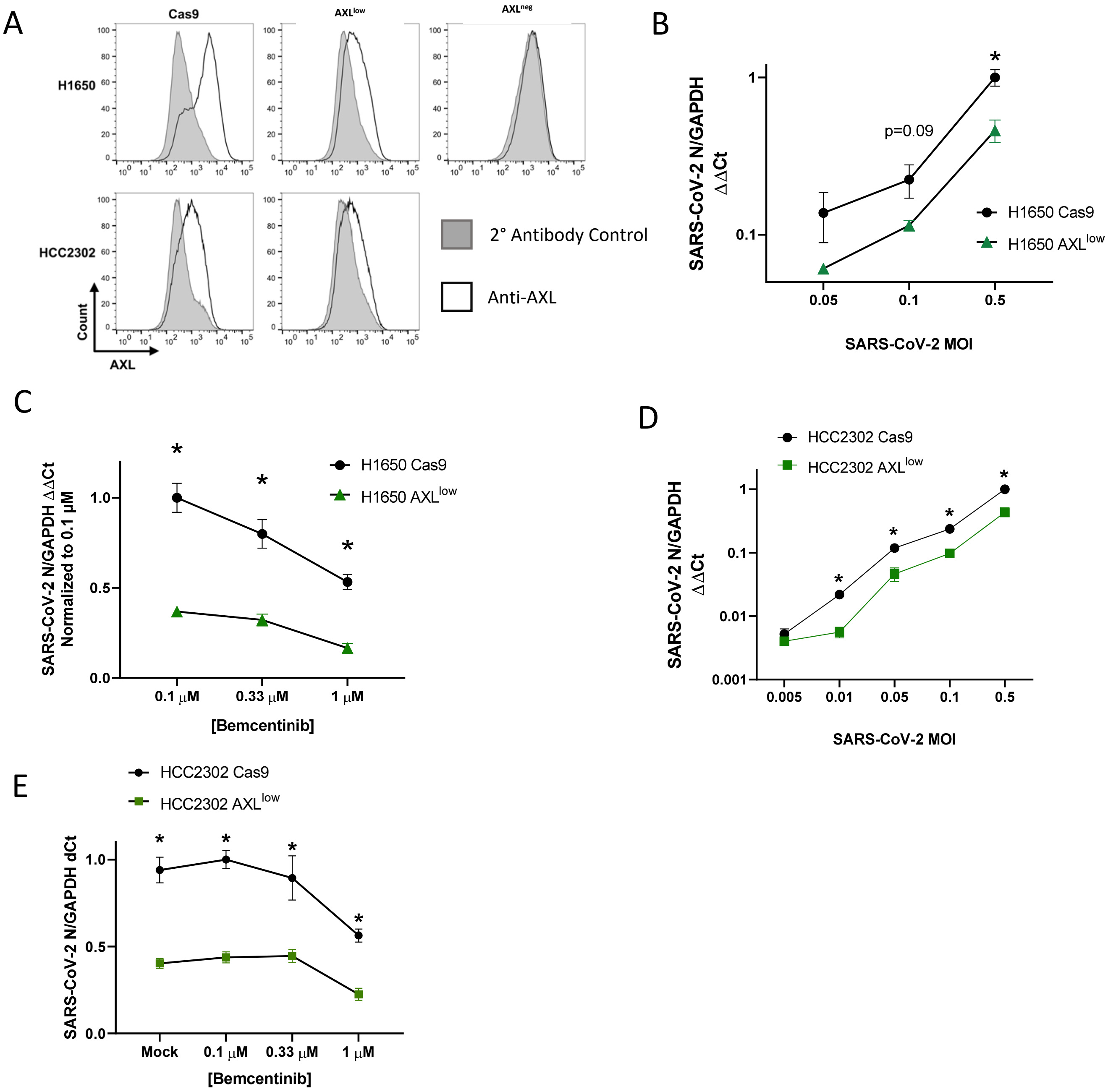

Supplemental Figure 6: AXL knockout reduces viral loads and ablates inhibition by bemcentinib. A) H1650 and HCC2302 AXL knockout cells were generated by lentiviral transduction of Cas9 and gRNA targeting AXL, followed by selection. These are designated “Bulk AXL^{low}” Shown are flow cytometry histograms depicting AXL surface staining (black) and secondary only background (grey), demonstrating complete loss of AXL expression in H1650 AXL^{neg}. **B)** H1650 AXL^{low} and H1650 Cas9 (parental) lines were challenged with SARS-CoV-2 at indicated MOIs for 24 hpi and viral loads assessed by RT-qPCR. **C)** H1650 parental and AXL^{low} lines were treated with indicated concentration of bemcentinib for 1 hour and subsequently challenged with SARS-CoV-2 (MOI = 0.5) and viral loads determine by RT-qPCR 24 hpi. **D-E)** As in B-C with HCC2302 cells.
Data are pooled from at least 3 independent experiments (**B**, **C**, **D**, **E**) or are representative of at least 3 experiments (**A**). Data represented as means ± SEM. Multiple t-tests were performed; asterisks represent $p < 0.05$.

S7: Mouse Hepatitis Virus

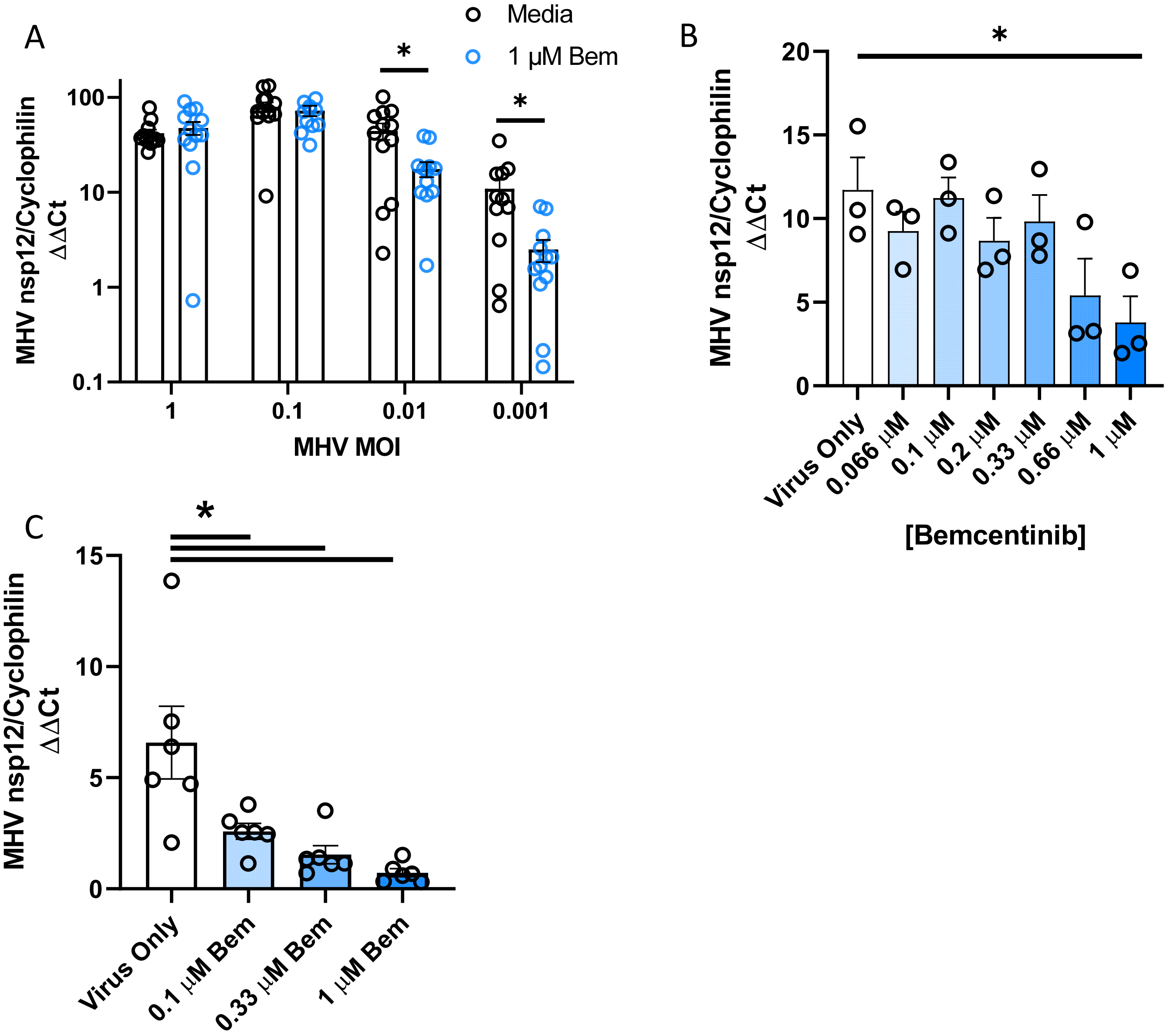

Supplemental Figure 7: A) Bone marrow derived macrophages from C57bl6/J mice were treated as indicated for 1 hour, challenged with MHV (stain A59) at the indicated MOI. Viral loads were assessed 24 hpi by RT-qPCR. **B)** BMDMs were treated with indicated concentrations of bemcentinib for 1 hour, infected with MHV (MOI = 0.001) for 24 hours and viral load assessed by RT-qPCR. **C)** As in B, MHV infection of peritoneal macrophages (MOI =0.001) treated with indicated concentrations of bemcentinib. Data shown are representative of 3 independent experiments. Data represented as means ± SEM. Student's t-test; asterisks represent $p < 0.05$.

Supplemental Figure 8: Author contributions, as defined by CRediT (Contributor Roles Taxonomy).

	Conceptualization	Methodology	Software	Validation	Formal Analysis	Investigation	Resources	Data Curation	Writing- Original Draft	Writing - Review and editing	Visualization	Supervision	Project Administration	Funding Acquisition
**D.B.**	■	■		■	■	■		■	■	■	■		■	
**H.V.E.**	■	■		■	■	■		■		■	■			
N.R.					■	■								
K.J.R.	■				■	■				■				
M.B.						■								
J.A.A.B		■				■				■				
R.A.R.C.						■								
B.G.							■							
T.S.			■		■			■						
E.C.						■								
D.M.		■			■	■	■							
G.G.										■				
H.H.		■			■	■	■							
J.M.	■						■							
J.B.L.	■	■				■	■					■		
**W.J.M.**	■	■		■	■	■	■	■	■	■		■	■	■
